# Who should we ask about mental health symptoms in adolescents with CFS/ME? Parent-child agreement on the revised children’s anxiety and depression scale

**DOI:** 10.1177/1359104521994880

**Published:** 2021-02-15

**Authors:** Teona Serafimova, Maria Loades, Daisy Gaunt, Esther Crawley

**Affiliations:** 1 Centre for Academic Child Health, Bristol Medical School, University of Bristol, Bristol, UK; 2 Department of Psychology, University of Bath, UK; 3 Royal United Hospital, Bath, UK

**Keywords:** Chronic fatigue syndrome, paediatric chronic fatigue syndrome, myalgic encephalomyelitis, parent child agreement, depression, anxiety

## Abstract

**Background:**

One in three adolescents with chronic fatigue syndrome/myalgic encephalomyelitis (CFS/ME) have mental health problems. Multi-informant perspectives are key to psychological assessment. Understanding parent-child agreement is crucial to accurate diagnosis, particularly where severe fatigue limits self-report.

**Methods:**

Agreement on the revised children’s anxiety and depression scale (RCADs) was assessed between parents and children with CFS/ME (*n* = 93) using Bland-Altman plots, cross tabulations and regression analyses.

**Results:**

Diagnostic thresholds were met more frequently based on child-report. Parent- and child-report had similar sensitivity and specificity on RCADS compared to gold-standard diagnostic interviews. Regression analysis found similar accuracy between both reports. For anxiety diagnoses, odds ratio (OR) for child-report was 1.10 (CI = 1.06–1.14), and 1.10 (CI = 1.05–1.14) for parent-report. For depression, OR for child report was 1.26 (CI = 1.11–1.43), while for parent-report is was 1.25 (CI = 1.10–1.41). For total score, OR for child-report was 1.10 (CI = 1.05–1.13) while OR for parent-report was 1.09 (CI = 1.05–1.13).

**Conclusions:**

Reasonable agreement was observed between parent- and child-report of mental health symptoms in paediatric CFS/ME. While parent-report can facilitate psychological evaluation in CFS/ME, this is not a substitute for a child’s own report.

## Introduction

Chronic fatigue syndrome/myalgic encephalomyelitis (CFS/ME) is a disabling condition which affects between 1% and 2% of the paediatric population (Crawley et al., 2011; Haines et al., 2005; Nijhof et al., 2011). Paediatric CFS/ME can involve a wide range of symptoms and have a profound impact on children’s functioning (Brigden et al., 2018; Loades, Crawley, et al., 2020; Loades, Vitoratou, et al., 2020; Parslow et al., 2017). Comorbid depression and/or anxiety is also common amongst children with CFS/ME (Bould et al., 2013; Loades et al., 2018). This is consistent with existing research suggesting an association between increased prevalence of mental health problems amongst children with existing chronic health problems (Brady et al., 2020). Approximately one-third of those with paediatric CFS/ME meet the criteria for a mental health problem (Loades, Read, et al., 2020). This is in excess of the prevalence in the wider paediatric population (Jane Costello et al., 2006). Co-morbid mental health problems are associated with lower school attendance and worse self-reported social functioning (Loades et al., 2018, 2019). There are challenges to establishing diagnoses of mental health disorders in the CFS/ME population due to symptom conflation, which particularly affects symptoms such as anergia, fatigue and sleep.

Current best practice recommends the use of psychiatric interview to diagnose mental health disorders in children (National Institute for Clinical Excellence, 2019). For paediatric depression, the gold standard interview is the Kiddie Schedule for Affective Disorders and Schizophrenia (KSADS) (Kaufman et al., 1997). However, in routine clinical practice, assessment often involves screening questionnaires, alongside unstructured clinical interviews. Thorough assessment of paediatric mental health also involves seeking multi-informant perspectives, most commonly from parents or carers. This can provide insight into contextual variations in mental health (De Los Reyes et al., 2015); differences may be observed at home compared to at school. This approach can also mitigate against both over- and under-reporting of symptoms, and self-report bias. In the context of paediatric CFS/ME, multi-informant approaches may be especially valuable as children may be too fatigued to engage in protracted mental health assessment needed to formulate a diagnosis.

### Parent-child agreement

Input from a parent or carer is valuable in paediatric mental health assessment. This is especially important where children lack insight into their behaviours or where illness prevents or limits the child from completing self-report measures. Understanding how parent proxy-report relates to a child’s responses is key to understanding the reliability of parental report. When parental and child responses are congruent, this is termed ‘parent-child agreement’. Parent-child agreement is highly variable, depending on multiple factors. One large meta-analysis of 341 studies demonstrated only low to moderate correlations between children and parents in relation to mental health symptoms (De Los Reyes et al., 2015). Recent evidence evaluating depression found that parents identified fewer symptoms than their adolescent children, meaning adolescents met the diagnostic criteria less frequently based on parental responses (Orchard et al., 2019). This is consistent with previous research showing that parental proxy-report identifies depressive symptoms less frequently than their child’s self-report (Eg et al., 2018; Orchard et al., 2017). Higher agreement has been demonstrated for externalising behaviours (Comer & Kendall, 2004; Hemmingsson et al., 2017; Orchard et al., 2019), which are often directly observable or occur in the home environment. This is true for both mental and physical health parameters.

Parent-child agreement is also affected by child and parent factors. Parental understanding of children’s mental health will depend on the extent to which the child feels able to verbalise their internal world. Other factors include co-morbid mental health diagnoses in the child (Hicks White & Snyder, 2018), the degree of parental involvement (Upton et al., 2008) and parental depression (Chi & Hinshaw, 2002). No clear association has been shown between parent-child agreement and age, gender or child functional limitation (Hemmingsson et al., 2017). Further, discrepancy between parent and child reports may provide greater insight into a child’s mental health than agreement, particularly depending on whether the child or parent cites a greater symptom burden.

When examining the revised children’s anxiety and depression scale (RCADS), and the shortened version RCADS-25, studies have found parent-child agreement to be moderate at best (Gormez et al., 2017; Muris et al., 2002), in a population with no underlying health problems. Consistent with work evaluating other rating scales, lower parent-child agreement has been observed for internalising symptoms measured on the RCADS (Ebesutani et al., 2011; Gormez et al., 2017; Muris et al., 2002). As such, correlations are highest for symptoms pertaining to separation anxiety disorder (Ebesutani et al., 2011; Gormez et al., 2017) and lowest for symptoms of generalised anxiety disorder (Ebesutani et al., 2011). However, interestingly, in the context of comorbid eating disorder, parents/carers gave higher anxiety and depression scores on the RCADS than children (Hicks White & Snyder, 2018). Agreement was also found to increase with increasing severity of the eating disorder.

No study thus far has evaluated the degree of parent-child agreement in relation to mental health symptoms in a population with paediatric CFS/ME. One study found reasonable parent-child agreement on two different self-report scales used to assess common CFS-like symptoms (Holtzman et al., 2018). However, neither scale specifically evaluated mental health. Where there was significant discrepancy, the difference in scores between parent and child were large (a 5-point difference on a 100-point scale). These discrepancies may be of clinical significance, which suggests that both parent and child input is of value.

Evaluating parent-child agreement with regards to mental health in paediatric CFS/ME is valuable for several reasons. Understanding the degree to which parental reports are consistent with children’s symptoms is important where children are too fatigued to complete full psychiatric interviews or questionnaires. If there is poor parent-child agreement, there may be a risk of missed diagnoses if only one informant is used. Alternatively, if agreement is high, there is a risk of redundancy and unnecessary effort. Parental insight into children’s mental health is also key as parents facilitate children’s access to specialist services (De Los Reyes et al., 2015). Parental concern has been shown to increase sensitivity of GP recognition of mental health disorders in children (Sayal & Taylor, 2004). Therefore, examining parent-child agreement on mental health symptoms on commonly used screening questionnaires is important. This has not previously been addressed in children and young people with CFS/ME, despite the high prevalence of mental health problems in this population.

We aimed to compare parent proxy report and adolescent child self-report (henceforth referred to as ‘child’ for clarity) of symptoms of common mental health problems on a screening questionnaire. Our study focused on adolescents, between 12 and 18 years old. Given existing research suggesting that parental proxy reports include fewer mental health symptoms than the child’s own report, we anticipated similar patterns would be observed in our data.

Our specific research questions were:

1. Do child- and parent-reports on the RCADS agree when comparing scores for the depression and anxiety subscales, and total scores?
  Hypothesis: We anticipated that agreement between parent- and child-report would be moderate at best.
2. Do children meet the diagnostic thresholds for mental health problems more frequently on parent- or self-report?
  Hypothesis: We expected that diagnostic thresholds would be met more frequently on the basis of child-report.
3. Does child or parent report on the RCADS more accurately predict diagnostic status for depression and anxiety on the gold standard (KSADS)?
  Hypothesis: We expected that child report would more accurately predict diagnostic status.

## Methods

This study involves retrospective analysis of data collected as part of the Wellbeing Study (Loades, Read, et al., 2020). This was a cross-sectional study conducted in a tertiary paediatric CFS/ME service. Participants completed a range of psychometric and other tests. However, we only analysed data from participants who completed the study measures of relevance here (e.g. both the RCADS and KSADS). (Loades, Read, et al., 2020).

### Participants

We recruited children attending an initial clinical assessment at a specialist paediatric CFS/ME service from September 2016 to April 2019. A total of 93 participants were recruited. Children were considered eligible if they met the following inclusion criteria: diagnosis of CFS/ME according to NICE (2007) criteria; age 12 to 18; and able to complete study assessments (i.e. a diagnostic interview and the screening questionnaires). Exclusion criteria included: fatigue explained by other diagnoses; unable to complete measures because of learning difficulties; and unable to complete structured diagnostic interview (e.g. insufficient English or severely affected).

### Measures

Demographic data was collected from medical records, including age and gender. In addition to the measures described below, participants also completed a range of routinely administered questionnaires including the Hospital Anxiety and Depression Scale, Chalder Fatigue Questionnaire and the SF-36 physical function subscale.

### RCADS

The revised children’s anxiety and depression scale (RCADS) was completed by both children and their parent separately. The RCADS is a 47-item self-report questionnaire that examines symptoms of depression and anxiety, based on the DSM-IV (American Psychiatric Association, 2000). Ten items pertain to the depression subscale and 37 items to the anxiety subscale. Each item is rated between 0 and 3, and scores are summed, with higher scores indicating greater symptomatology. The RCADS has strong psychometric properties (Ebesutani et al., 2011) and is routinely used in Child and Adolescent Mental Health Services (CAMHS) in the UK. There is a version for child self-report (RCADS-C) and a version for parents as proxy informants (RCADS-P). In this study, children and parents completed their respective versions on the same day. Cronbach’s alpha for psychological measures used in this study were >0.8, indicating acceptable internal consistency.

### KSADS

The KSADS (Kiddie Schedule for Affective Disorders and Schizophrenia) is a semi-structured interview validated for the early diagnosis of mood disorders (including depression, bipolar disorder and anxiety) in children (aged 6–18 years old). It is the gold standard instrument for diagnosis of depression in paediatric populations and is recommended by NICE (National Institute For Clinical Excellence, 2019). The KSADS was administered by interview, either via Skype, telephone or face to face. Interviews were conducted by members of the research team who had completed a 2-day training course. Both children and their parents were interviewed, either together, or children followed by parents. The children decided whether they would prefer to be interviewed alone or together. Where interviews were conducted separately, information was integrated by the interviewer before diagnostic decisions were made.

### Procedure

Potential participants were informed of the study by their assessing clinician at the specialist CFS/ME service during their initial clinical assessment and provided with an information sheet. Those who were interested in participating provided consent to be contacted by the research team. Following discussion with the research team, those who agreed to participate provided written consent. Where participants were under the age of 16, the parent or guardian gave consent on their behalf. The child provided written assent. Irrespective of the child’s age, parents were also asked for consent for their own participation in the study.

Once consent had been obtained, participants and their parents completed the study assessments, including the RCADS and RCADS-P, either online via REDCAP (Research Electronic Data Capture) hosted at the University of Bristol (Harris et al., 2009, 2019) or pen-and-paper.

Research assistants provided support with questionnaire completion where required (for example, reading items out loud and recording participant responses where their fatigue and concentration difficulties precluded doing this independently). The KSADS interview was then conducted via Skype, telephone or in person, according to participant preference. Children were given the option of being interviewed with parents or separately. Most (*N* = 69, 75.8%) chose to be interviewed together with their parent(s)/guardian(s). The remaining children (*N* = 22, 24.2%) were interview alone. Therefore, summary ratings only were generated, rather than separate ratings based on a child interview and a parent interview, as well as a summary integrated rating.

### Ethical approval

NHS research ethics committee (16/SW/036), University of Bath Department of Psychology Research Ethics Committee (16-203) and relevant research and development department approval was obtained.

### Data analyses

Data were analysed using IBM SPSS Statistics 24. Descriptive statistics were used to describe the sample characteristics (e.g. participant age, gender) and the variables of interest (child self-reported anxiety and depression, parent proxy-reported anxiety and depression). Tests for normality were conducted and these informed the choice of parametric versus non-parametric tests.

RQ1: Bland-Altman plots were constructed to describe agreement between parent- and child- report on the anxiety subscale, depression subscale and total score.
RQ2: Cross tabulations were used to establish how many participants were correctly and incorrectly classified as depressed and/or anxious on the RCADS self-report and parent report, where the KSADS provided a diagnostic benchmark. A cut-off of >15 was used on the depression subscale to indicate probable clinical depression. A cut-off of >38 on the anxiety subscale was used to indicate probably anxiety. A cut-off of >48 for the total RCADS score was used to indicate distress. These cut offs were determined from Receiver Operation Characteristic curves reported elsewhere (Loades, Read, et al., 2020).
RQ3: Logistic regression was completed to evaluate the extent to which parent- or child-report predicted a mental health problem diagnosed using the gold-standard KSADS.

## Results

During the recruitment period, 289 potentially eligible patients attended an initial assessment at the recruitment site. The majority gave consent to be contacted by the team (*n* = 214) and 177 potential participants were successfully contacted. A total of 107 participants initially consented to participate in the study although 15 subsequently withdrew. A total of 93 young people with CFS/ME took part in this study, mean age 15.1 (SD = 1.58). Most participants were female (*N* = 59, 63%) and British (*N* = 80, 86%). See Table 1 for baseline characteristics.

**Table 1.** Baseline characteristics of participants.

	*N* (%)	
Gender		
Female	59 (63.4)	
Ethnic origin		
British	80 (86)	
Any other white background	2 (2.2)	
Pakistani	1 (1.1)	
Other ethnic group	1 (1.1)	
Missing	9 (9.7)	
	Range	Mean (SD)
Age (years)	12–18	15.1 (1.58)
Chalder fatigue Questionnaire^a^	11–33	26 (4.5)
SF36 – PFS^b^	0–100	50 (25.5)

*Note*. ^a^*Chalder Fatigue Questionnaire* – participants can score a maximum of 33. A higher score indicates worse fatigue.

b *SF36-PFS – Short Form (36) – physical functioning scale –* participants can score a maximum of 100. A higher score indicates better physical functioning.

**Figure 1.**
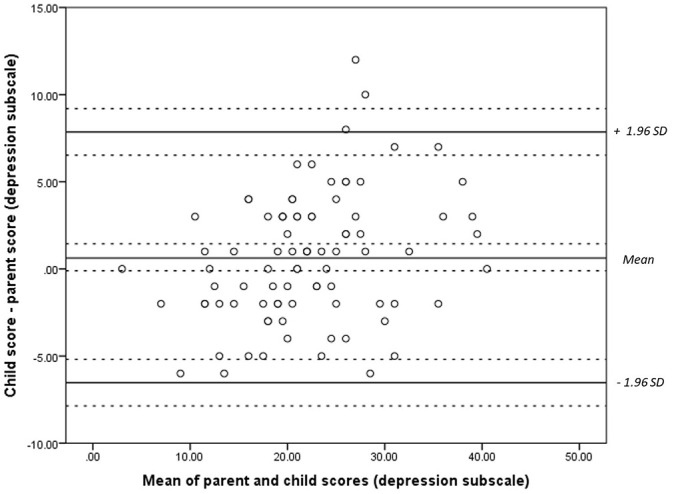
Bland-Altman plot comparing child and parental responses on the depression subscale of the RCADS. Dashed lines represent the limits of agreement (LOA) of the mean and confidence intervals.

**Figure 2.**
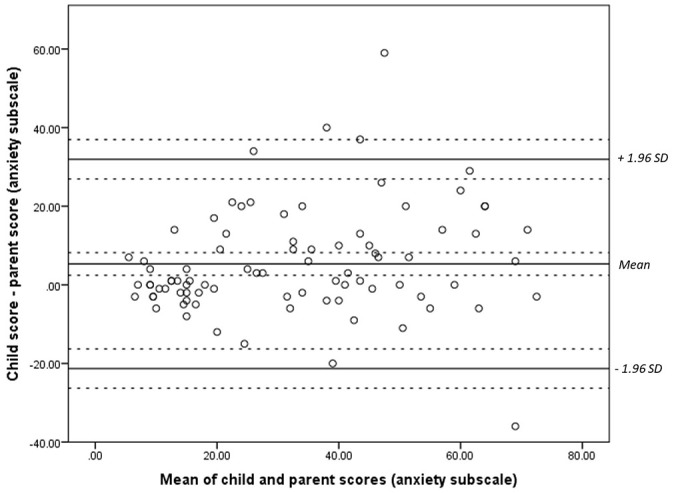
Bland-Altman plot comparing child and parental responses on the anxiety subscale of the RCADS. Dashed lines represent the limits of agreement (LOA) of the mean and confidence intervals.

**Figure 3.**
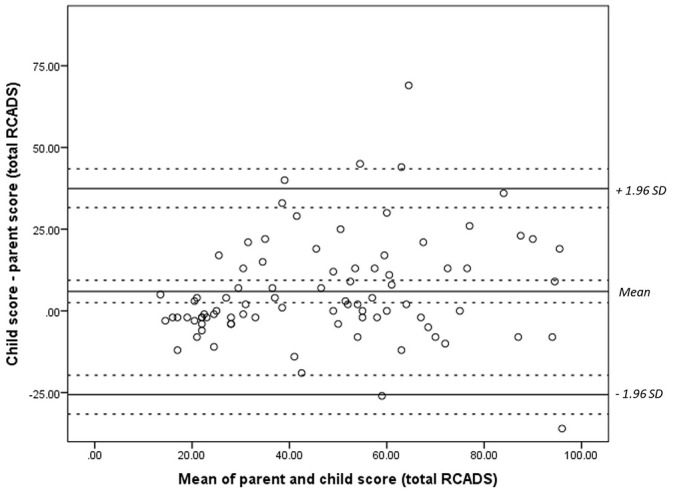
Bland-Altman plot comparing child and parental responses for generalised distress on the RCADS. Dashed lines represent the limits of agreement (LOA) of the mean and confidence intervals.

Bland-Altman plots were constructed comparing paired measurements of child- and parent- report on the RCADS (Figures 1–3). The plots show differences between parent and child scores against the means of their scores. Test for normality were conducted prior to the construction of these plots. No transformation of the data was required. Confidence intervals and limits of agreement (LOA) were calculated taking the data structure into account. We were unable to assess repeatability given the values measured are likely to vary.

For the depression subscale (Figure 1), values were distributed randomly, with little association between mean scores and the difference in scores given by parents and children. For the anxiety subscale (Figure 2) and total score (Figure 3), greater variability was observed at higher total scores.

Most of the difference values, for subscales and total scores occurred within the range of the mean ± 1.96 SD. Differences in parent-child scores exceeded the upper confidence interval in four cases for the anxiety subscale and total score, and in three cases for the depression subscale. For the depression subscale, this was 0.7 ± 3.7 (range = −6.6 to 8.0 points). For the anxiety subscale this was 5.3 ± 13.6 (range = −21 to 32). For total score, this was 6.0 ± 16.1 (−25.6 to –38.0). These ranges are all beyond what would be deemed clinically acceptable.

On the RCADS-C, 38 (40.9%) of children met thresholds for depression, 38 met thresholds for anxiety disorders (40.9%) and 49 (52.7%) met thresholds for any mental health disorder. Lower numbers were identified on the RCADS-P (see Table 2) for all subscales.

**Table 2.** Mean scores on RCADS subscales and proportion of participants exceeding the diagnostic threshold based on child or parent report.

Child report	Mean (SD)	Parent report	Mean (SD)
RCADS-C depression	15 (5.4)	RCADS-P depression	14 (4.8)
RCADS-C anxiety	35 (20.7)	RCADS-P anxiety	30 (18.7)
RCADS-C total	50 (24.7)	RCADS-P total	44 (22.4)
Proportion above cut off			
Child report	*N* (%)	Parent report	*N* (%)
Depression subscale score > 15	38 (40.9)	Depression subscale score > 15	29 (31.2)
Anxiety subscale score > 38	38 (40.9)	Anxiety subscale score > 38	30 (32.3)
Total score > 48	49 (52.7)	Total score > 48	36 (38.7)

*Note*. RCADS-C = revised children’s anxiety and depression scale – child form; RCADS-P = revised children’s anxiety and depression scale – parent form.

Scores on the depression subscale of the RCADS-C accurately predicted a diagnosis of depression on the KSADS in 69.9% of children (Table 3). Scores on the depression subscale of the RCADS-P accurately predicted a diagnosis of depression in 75.3% of children. Higher accuracy was seen for anxiety and mood disorders in general. When considering the anxiety subscale of the RCADS-C, scores accurately predicted an anxiety disorder in 76.3% of cases. For the RCADS-P, 79.6% of cases were accurately predicted. When evaluating total scores on the RCADS-C, scores accurately predicted a diagnosis of any mood disorder in 78.5% of children. Similarly, when using the RCADS-P, 75.2% of cases were correctly identified.

**Table 3.** Diagnosis of depression, anxiety or generalised distress made using screening RCADS compared to diagnosis using KSADs.

		Child-report over threshold for depression^b^		Parent-report over threshold for depression^b^	
Confirmed mental health diagnosisa		No (%)	Yes (%)	No	Yes
	No	47 (50.5%)	20 (21.5%)	54 (58.1%)	13 (14.0%)
	Yes	8 (8.6%)	18 (19.4%)	10 (10.8%)	16 (17.2%)
		Child-report over threshold for anxiety^c^		Parent-report over threshold for anxiety^c^	
		No	Yes	No	Yes
	No	47 (50.5%)	14 (15.1%)	53 (57.0%)	10 (10.8%)
	Yes	8 (8.6%)	24 (25.8%)	9 (9.7%)	21 (22.6%)
		Child-report of distress (over threshold for anxiety or depression)^d^		Parent-report of distress (over threshold for anxiety or depression)^d^	
		No	Yes	No	Yes
	No	38 (40.9%)	6 (6.5%)	43 (46.2%)	14 (15.1%)
	Yes	14 (15.1%)	35 (37.6%)	9 (9.7%)	27 (29.0%)

*Note*. KSADs = Kiddie-schedule for affective disorders and schizophrenia; RCADS = revised children’s anxiety and depression scale.

a Relevant diagnosis (depression and/or anxiety) made through KSADS interview.

b RCADS score > 15 on depression subscale.

c RCADS score > 38 on anxiety subscale.

d RCADS score > 48 on total RCADS score.

Item level agreement was assessed using Cohen’s kappa (Supplemental Appendix 1). Item-level agreement was generally slight to moderate (range = 0.16–0.66). Agreement was moderate for observable symptoms (range = 0.34–0.61).

Logistic regression was carried out to evaluate whether child or parent-proxy report more accurately predicted diagnostic status for depression, anxiety disorders and any mental health disorder identified on the KSADS. The model included age, gender and child- or parental scores on each subscale (depression, anxiety and total score). Child and parent report predicted depression diagnosed by KSADS with similar accuracy for both subscales and total score.

For depression, odds ratios (OR) for child report was 1.26 (*p* < .001, CI = 1.11–1.43), while that for parental report was 1.25 (*p* < .001, CI = 1.10–1.41). When evaluating anxiety diagnoses, OR for child report was 1.10 (*p* < .001, CI = 1.06–1.14). For parental report, it was 1.10 (*p* < .001, CI = 1.05–1.14). When examining total scores, OR for child report was 1.10 (*p* < .001, CI = 1.05–1.13). OR for parental report was 1.09 (*p* < .001, CI = 1.050–1.13).

## Discussion

This is the first study to evaluate, in detail, parent-child agreement with regards to mental health symptoms in paediatric CFS/ME. Parent-child agreement was generally reasonable. Diagnostic thresholds were met more frequently based on child-report. The RCADS showed adequate sensitivity and specificity in the prediction of mental health disorders, irrespective of who completed them. Child-report on the RCADS predicted diagnoses on the KSADS with improved accuracy compared to parent-report.

Previous research has shown parent-child agreement to highly variable. Poor agreement has been shown for diagnostic tests for depression (Orchard et al., 2019) whereas high agreement has been shown when evaluating mental health symptoms in a cohort of children with eating disorders (Hicks White & Snyder, 2018). The factors contributing to increased agreement in the context of both eating disorders and paediatric CFS/ME are multifactorial and may include, but are not limited to, increased dependency between parents and children, increased parental anxiety/vigilance, and increased communication between parents and children. One possible explanation for reasonable parent-children agreement in the context of paediatric CFS/ME may be secondary to increased observation of children by their parents . This is particularly relevant for those children who are significantly limited by CFS/ME and unable to attend school or other activities. Increased proximity may also facilitate greater closeness and openness between children and their parents. Future research could investigate this.

Given paediatric CSF/ME is characterised by severe fatigue which limits activity, avoiding protracted assessments such as the KSADs may be necessary. While shorter assessments cannot provide the same level of detailed information into a child’s mental health, this may represent an appropriate compromise for some patients. In settings where children are extremely fatigued, avoiding additional burdens on the patient is crucial. Here, parental report could be used to gain an initial insight into the mental health status of the child, using a questionnaire such as the RCADS-P. This can prompt further, more thorough assessment at a later time.

### Strengths and weaknesses

This study benefits from recruiting children and parents from a specialist NHS service for CFS/ME. Accurate assessment of mental health symptoms in children with CSF/ME is highly relevant, given the increased prevalence of mental health disorders in this population. Severe fatigue secondary to CSF/ME can prevent children from undertaking assessment, making parental proxy report even more valuable. Understanding parent-child agreement in this setting will facilitate appropriate interpretation of proxy reports in clinical settings. By assessing agreement in common, diagnostic tools and interviews, this study will be relevant to many clinicians working in CFS/ME care.

While our sample is relatively large, recruitment from a specialist CFS/ME service may result in an overrepresentation of severe cases, who, in turn, may also have a higher burden of mental illness. Given that parents facilitate access to specialist services, families of children seen in a tertiary referral centre may have greater insight into their children’s health. A substantial proportion of those initially invited to participate declined, meaning our sample may not accurately represent the clinical population at the specialist service. Those who participated may represent children who are more severely affected by mental health problems or those whose families recognise symptoms of mental illness in their children. As a result, our results may not be generalisable to the general CFS/ME population. Further, if our assumption regarding parental engagement is true, this may result in increased parent-child agreement.

Our study included participants across a broad range of ages, at different developmental stages. This is likely to influence their day-to-day activities, levels of independence as well as their approach to psychometric testing. These factors may influence the degree of parent-child agreement observed. Older adolescents (those who are 17 or 18) may be more independent and have broader support networks, which may result in reduced parent-child agreement. Conversely, younger children may spend more time with their family, meaning any mental health concerns may be more apparent. Although this may be true of a population with no comorbidities, the relationship between these factors is likely to be nuanced in the context of chronic illness. It is also relevant to note that our study did not include children younger than 12 years old, meaning our results cannot be generalised to a primary school aged population.

The majority of children in our study completed the KSADS interview together with their parents. Children may give more socially desirable answers, particularly surrounding sensitive or difficult topics, which may result in some symptoms being underreported. While interviewing children alone would be of value, this may not be feasible with younger children. Child self-report on the RCADS may provide greater insight into the child’s personal experience of their psychological wellbeing and would not be modified by parental presence, as was the case at interview for some participants. Children may find interviews, particularly regarding their thoughts and feelings, challenging. For those children, expressing this through a questionnaire may be more straightforward and may yield more meaningful information than would be gained through an interview. That said, questionnaire responses would inherently provide less detailed response from children.

We did not collect data regarding the baseline characteristics of the participants’ parents. We also did not evaluate other background factors which may have been of relevance such children’s comorbidities or parental mental health problems.

Evaluating psychosocial factors affecting the health of children and young people with chronic illness is increasingly recognised as important (Anderson et al., 2020; Mattson & Kuo, 2019). Although gold-standard methods such as the KSADs are valuable and provide a comprehensive assessment of the patient’s mental wellbeing, this is not always pragmatic and may be too labour intensive to be included in routine clinical settings. Hence, patient reported outcomes, such as the RCADS, represent valuable methods of assessing such issues and provide a highly personal insight into a child’s wellbeing. Inclusion of caregiver reports is also valuable in facilitating parental engagement and developing a more holistic insight into the child’s wellbeing. Further, involving caregivers in the screening process may reduce stigma and enhance vigilance towards psychosocial problems. This is particularly important given parents can act as gatekeepers to secondary care.

## Conclusion

This study provides important insight to those assessing mental health symptoms in the paediatric CFS/ME population. Consistent with previous literature, mental health disorders are common within this population, highlighting the need for clinician vigilance towards these issues. Moderate parent-child agreement suggests that parental proxy report is useful in this context. Parental report may provide a reasonable insight into a child’s mental health where fatigue precludes extensive mental health assessment. However, this should not act as a substitute for a child’s own report of their symptoms, where this can be obtained. Ultimately, clinicians will need to tailor assessment of mental health problems in this population to what is acceptable to the child as well as what is pragmatic. Future research should investigate whether similar agreement is seen in younger children, particularly those of primary school age. This is especially relevant given interview-style psychological assessments may be more challenging in a younger age group. Additional investigation of the factors that predict parent-child discrepancy may also be of value in highlighting circumstances where parent-proxy report may be less helpful.

## Supplemental Material

sj-pdf-1-ccp-10.1177_1359104521994880 – Supplemental material for Who should we ask about mental health symptoms in adolescents with CFS/ME? Parent-child agreement on the revised children’s anxiety and depression scale Click here for additional data file. Supplemental material, sj-pdf-1-ccp-10.1177_1359104521994880 for Who should we ask about mental health symptoms in adolescents with CFS/ME? Parent-child agreement on the revised children’s anxiety and depression scale by Teona Serafimova, Maria Loades, Daisy Gaunt and Esther Crawley in Clinical Child Psychology and Psychiatry
