# Supplementary material for: Who should we ask about mental health symptoms in adolescents with CFS/ME? Parent-child agreement on the revised children’s anxiety and depression scale
Source: Clin Child Psychol Psychiatry. 2021 Feb 15;26(2):367–80. doi: 10.1177/1359104521994880 (PMC8172254; doi:10.1177/1359104521994880)
Supplement: sj-pdf-1-ccp-10.1177_1359104521994880 – Supplemental material for Who should we ask about mental health symptoms in adolescents with CFS/ME? Parent-child agreement on the revised children’s anxiety and depression scale [file sj-pdf-1-ccp-10.1177_1359104521994880.pdf]

Appendix 1

RCADS Item	Kappa	Significance (95% CI)	Observable symptom?
**Major Depression subscale**			
2. I feel sad or empty	0.34	<0.001 (0.17-0.51)	
6. Nothing is much fun anymore	0.21	0.001 (0.07-0.36)	
11. I have trouble sleeping	0.66	<0.001 (0.53-0.78)	X
15. I have problems with my appetite	0.42	<0.001 (0.28-0.57)	X
19. I have no energy for things.	0.39	<0.001 (0.23-0.56)	X
21. I am tired a lot.	0.61	<0.001 (0.44-0.79)	X
25. I cannot think clearly	0.36	<0.001 (0.20-0.52)	
29. I feel worthless	0.28	<0.001 (0.12-0.43)	
40. I feel like I don’t want to move	0.30	<0.001 (0.15-0.45)	
47. I feel restless	0.28	<0.001 (0.14-0.43)	X
**Anxiety related items**			
1. I worry about things	0.41	<0.001 (0.25-0.56)	
3. When I have a problem, I get a funny feeling in my stomach	0.57	<0.001 (0.44-0.71)	
4. I worry when I think I have done poorly at something	0.38	<0.001 (0.23-0.52)	
5. I would feel afraid of being on my own at home	0.41	<0.001 (0.24-0.58)	X
7. I feel scared when I have to take a test	0.44	<0.001 (0.31-0.58)	
8. I feel worried when I think someone is angry with me	0.37	<0.001 (0.23-0.51)	
9. I worry about being away from my parents	0.43	<0.001 (0.29-0.58)	
10. I get bothered by bad or silly thoughts or pictures in my mind.	0.24	0.001 (0.09-0.39)	
12. I worry that I will do badly at my school work	0.47	<0.001 (0.34-0.61)	
13. I worry that something awful will happen to someone in my family	0.46	<0.001 (0.31-0.61)	
14. I suddenly feel as if I can't breathe when there is no reason for this	0.48	<0.001 (0.31-0.64)	X
16. I have to keep checking that I have done things right (like the switch is off, or the door is locked)	0.31	<0.001 (0.17-0.46)	
17 I feel scared if I have to sleep on my own.	0.45	<0.001 (0.25-0.66)	X
18. I have trouble going to school in the mornings because I feel nervous or afraid	0.46	<0.001 (0.31-0.61)	X
20. I worry I might look foolish	0.38	<0.001 (0.24-0.53)	

22. I worry that bad things will happen to me	0.35	<0.001 (0.20-0.50)	
23. I can't seem to get bad or silly thoughts out of my head.	0.23	0.005 (0.07-0.40)	
24. When I have a problem, my heart beats really fast.	0.43	<0.001 (0.29-0.58)	
26. I suddenly start to tremble or shake when there is no reason for this	0.34	<0.001 (0.19-0.49)	X
27. I worry that something bad will happen to me	0.31	<0.001 (0.17-0.45)	
28. When I have a problem, I feel shaky	0.31	<0.001 (0.16-0.45)	
30. I worry about making mistakes	0.49	<0.001 (0.35-0.62)	
31. I have to think of special thoughts (like numbers or words) to stop bad things from happening	0.34	<0.001 (0.12-0.57)	
32. I worry what other people think of me	0.44	<0.001 (0.30-0.58)	
33. I am afraid of being in crowded places (like shopping centres, the movies, buses, busy playgrounds)	0.35	<0.001 (0.21-0.49)	X
34. All of a sudden I feel really scared for no reason at all.	0.36	<0.001 (0.20-0.51)	
35. I worry about what is going to happen.	0.16	0.016 (0.01-0.31)	
36. I suddenly become dizzy or faint when there is no reason for this	0.43	<0.001 (0.29-0.57)	X
37. I think about death	0.57	<0.001 (0.42-0.73)	
38. I feel afraid if I have to talk in front of my class	0.37	<0.001 (0.23-0.51)	
39. My heart suddenly starts to beat too quickly for no reason.	0.64	<0.001 (0.50-0.77)	
41. I worry that I will suddenly get a scared feeling when there is nothing to be afraid of	0.30	<0.001 (0.14-0.47)	
42. I have to do some things over and over again (like washing my hands, cleaning or putting things in a certain order)	0.43	<0.001 (0.26-0.59)	X
43. I feel afraid that I will make a fool of myself in front of people.	0.37	<0.001 (0.23-0.51)	
44. I have to do some things in just the right way to stop bad things from happening	0.17	0.023 (0.02-0.32)	
45. I worry when I go to bed at night	0.30	<0.001 (0.15-0.45)	
46. I would feel scared if I had to stay away from home overnight	0.44	<0.001 (0.27-0.61)	x
